# Multilayer Graphene with Chemical Modification as Transparent Conducting Electrodes in Organic Light-Emitting Diode

**DOI:** 10.1186/s11671-017-2009-9

**Published:** 2017-04-05

**Authors:** Yilin Xu, Haojian Yu, Cong Wang, Jin Cao, Yigang Chen, Zhongquan Ma, Ying You, Jixiang Wan, Xiaohong Fang, Xiaoyuan Chen

**Affiliations:** 1 School of Materials Science and Engineering, Shanghai University, Shanghai, 200444 China; 2 Thin Film Optoelectronic Technology Center, Shanghai Advanced Research Institute, Chinese Academy of Sciences, Shanghai, 201210 China; 3 Key Laboratory of Advanced Display and System Application, Shanghai University, Shanghai, 200072 China; 4 Department of Physics, Shanghai University, Shanghai, 200444 China; 5 School of Physical Science and Technology, Shanghai Tech University, Shanghai, 201210 China

**Keywords:** Multilayer-doped graphene, Pattern, OLEDs

## Abstract

Graphene is a promising candidate for the replacement of the typical transparent electrode indium tin oxide in optoelectronic devices. Currently, the application of polycrystalline graphene films grown by chemical vapor deposition is limited for their low electrical conductivity due to the poor transfer technique. In this work, we developed a new method of preparing tri-layer graphene films with chemical modification and explored the influence of doping and patterning process on the performance of the graphene films as transparent electrodes. In order to demonstrate the application of the tri-layer graphene films in optoelectronics, we fabricated the organic light-emitting diodes (OLEDs) based on them and found that plasma etching is feasible with certain influence on the quality of the graphene films and the performance of the OLEDs.

## Background

Transparent conductive materials play a significant role in optoelectronic devices such as solar cells [1–6], sensors [7–10], and organic light-emitting diodes (OLEDs) [11–13] and have attracted wide attention from the research community. The most conventionally used transparent conductive material is indium tin oxide (ITO), which has high optical transparency and electrical conductivity [14]. Nevertheless, the increasing cost of indium, brittleness, and photoelectric attenuation due to indium diffusion limited the development of the OLEDs. Graphene, as a member of the two-dimensional material community, has excellent photoelectric performance, such as ultra-high carrier mobility and transparency, which shows tremendous potential to replace ITO as transparent conductive electrodes in photoelectric devices [15, 16]. The common method of preparing large-area graphene films is through chemical vapor deposition (CVD) on metallic substrates, which typically contains the transfer of the as-grown graphene onto target substrates for further device fabrication. However, the quality of graphene is greatly influenced by the growth conditions, and the high sheet resistance is still caused by the polycrystallinity, wrinkles, and impurities introduced during the transfer process [17–19]. It has attracted wide attention to further improve the conductivity of graphene films on the premise of ensuring high transmittance and achieving the application of graphene films as the anodes of the OLEDs. Yu Wang et al. [20] reported that they explored the application of laminated graphene as the transparent electrodes in organic solar cells. Chen Nie et al. [21] reported that they used vinylidene chloride/acrylic ester copolymer (OA)/dichloromethane solution to modify graphene and reduce the sheet resistance to 300 Ω/□. Moreover, they utilized the graphene/PEDOT:PSS composite transparent conductive films as the anodes to fabricate flexible blue OLED devices with high current efficiency. However, the conductivity of graphene films decreases over time as the OA doping is not stable; it is difficult to satisfy the needs of industrial application. Jaehyun Moon et al. [22] recently reported that the graphene patterning using laser or plasma methods turned out to be problematic and could not preserve graphene quality. In this article, we developed a new method of preparing multilayer graphene with the chemical modification, which can improve its sheet resistance to 150 Ω/□ with the optical transmittance above 91% in the visible spectrum. We explored the influence of the patterning process using plasma on the graphene transparent electrodes. In addition, we prepared OLED devices based on the graphene anodes, and achieved reasonable luminous efficiency.

## Methods

### Preparation of Single-Layer Graphene Films

Single-layer graphene (SLG) were synthesized by CVD on a 25 μm polishing copper foil (99.999%, purchased from Alfa Aesar). The foils were annealed at 1000 °C in H_2_ atmosphere for 3–10 min in a tube furnace. Then, the source gas of CH_4_ was infused with a flow rate of 0.5–10 sccm by keeping the same temperature for 3–10 min. Finally, the copper foils were rapidly cooled to room temperature. The graphene films grown on the surface of the foils were transferred onto the target substrates by the following procedure:

1. The solution of polymethylmethacrylate (PMMA) in anisole was spin-coated on the copper foils with graphene for 30–60 s at 3000–6000 rpm (the thickness of PMMA films is about 200 nm).
2. The samples of Cu/graphene/PMMA were floated on FeCl_3_ aqueous solution to remove the copper foils.
3. The PMMA/graphene film was washed with deionized water and moved onto the glass substrate.
4. Finally, the PMMA/graphene films were immersed in acetone to remove the PMMA.

### Preparation of the Tri-Layer Graphene Film with Chemical Modification

**Fig. 1:**
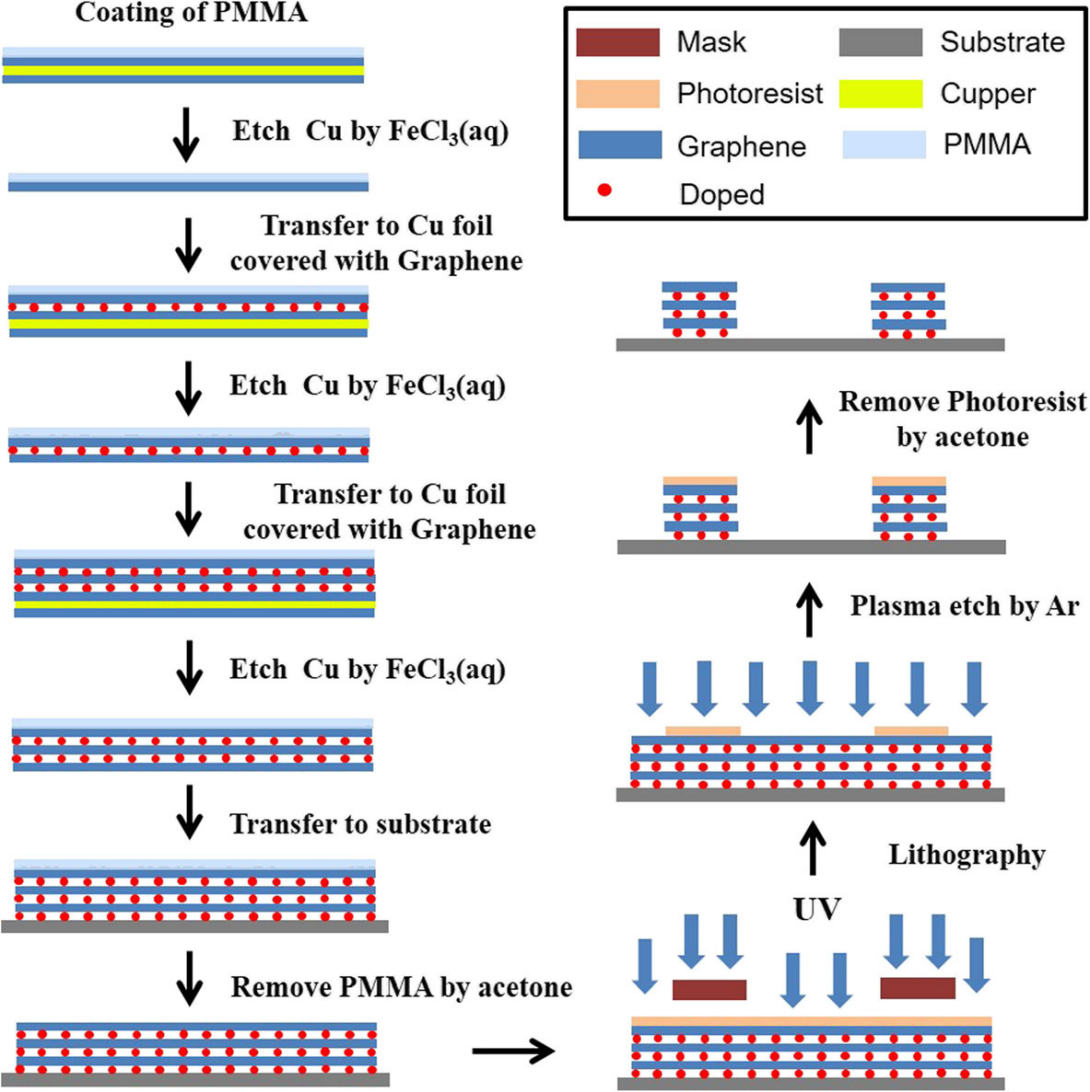
Schematic of the procedure on preparing the doped multilayer graphene film

The procedure used to prepare the tri-layer graphene films is schematically illustrated in Fig. 1. After being rinsed with deionized water for several times, the PMMA/graphene films were floated on the surface of the doping reagent for a few minutes. We chose 20 mM AuCl_3_ and HCl solution with the volume fraction of 20% as doping reagent. Then, we use the copper foils with graphene to pick up the PMMA/graphene from the dopant to form the PMMA/double-layer doped graphene/copper foils. After the dissolution of the copper, the multilayer graphene films with chemical modification can be obtained by repeating the above steps.

### Fabrication and Measurement of OLEDs

The tri-layer-doped graphene film was patterned by photolithography technology and etching. OLED devices were fabricated with a structure of graphene/*N*,*N*′-diphenyl-*N*,*N*′-bis (1-naphthyl)-(1,1′-biphenyl)-4,4′-diamin (NPB) (60 nm)/tris-(8-hydroxyquinoline) aluminum (Alq3) (60 nm)/lithium fluoride (LiF) (1 nm)/Al (80 nm), where NPB was used as the hole transport material, Alq3 was used as both the host material and the electron transport material, and LiF and Al were used as the electron inject material and the cathode material, respectively.

To characterize the graphene films, field-emission scanning electron microscope (SEM, FEI Quanta 600), transmission electron microscope, and atomic force microscope (NT-MDT) were used to examine the surface morphology. Raman measurements were performed using Thermo Scientific DXR Raman microscope spectrometer with a laser wavelength of 532 nm. For sheet resistance measurements, we used a semiconductor analyzer (Agilent, B1500A) combined with a four-probe station (CASCADE, alessi REL-4800). The optical transmittance in the wavelength range of 300–1100 nm was obtained by a PV Measurements QEX10. The current–voltage (*I*–*V*) characteristics of the fabricated OLEDs were measured with an experimental setup including a Keithley 2400 source meter. A spectroradiometer (PR750) was also employed to measure the electroluminescence spectrum of the 3 × 3 mm^2^ emitting area of the devices. The reference OLEDs with the same layer structures, except that the graphene film was replaced by a conventional ITO layer (20 Ω/□), were also fabricated for comparison.

## Results and Discussion

### Characterization of the Graphene Films

**Fig. 2:**
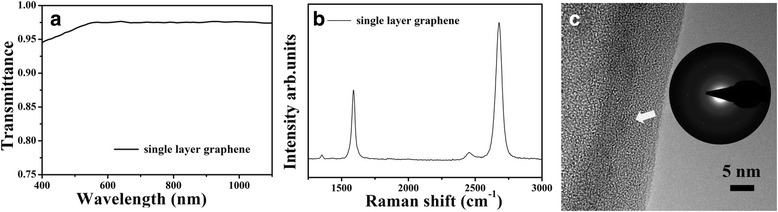
**a** Transmittance spectra. **b** Raman spectrum. **c** TEM image of the single-layer graphene

Figure 2a shows the transmittance of the SLG in the range of 300–1100 nm. We measured the transmittance spectrum of the SLG after transferring it onto the quartz glass substrate. The influence of the substrate has been removed from the results. The transmittance of the SLG at 550 nm is 97.4%, close to the theoretical value of 97.7%, indicating the single-layer feature of the graphene film. Raman spectrum is a quite vigorous tool to characterize the defects and layer number of the graphene films. Figure 2b shows the Raman spectrum of the graphene film, which involves two peaks of the G band and 2D band at 1590 and 2680 cm^−1^, respectively. The intensity ratio of *I*_2D_/*I*_G_ ≈ 2 indicating the graphene film is single layer. The peak of D band at 1352 cm^−1^ is exceptionally weak, showing high quality of the SLG. Transmission electron microscopy (TEM) was also carried out to confirm the quality of the graphene. From Fig. 2c, we can see that a regular hexagon is obvious, and there is only one set of diffraction pattern. The sheet resistance of SLG is up to 850 Ω/□ due to the polycrystalline nature of the graphene. The above result shows the high quality of the SLG film, but it is unfit for device application because of its high sheet resistance.

### Characterization of the Tri-Layer Graphene Film with Chemical Modification

**Fig. 3:**
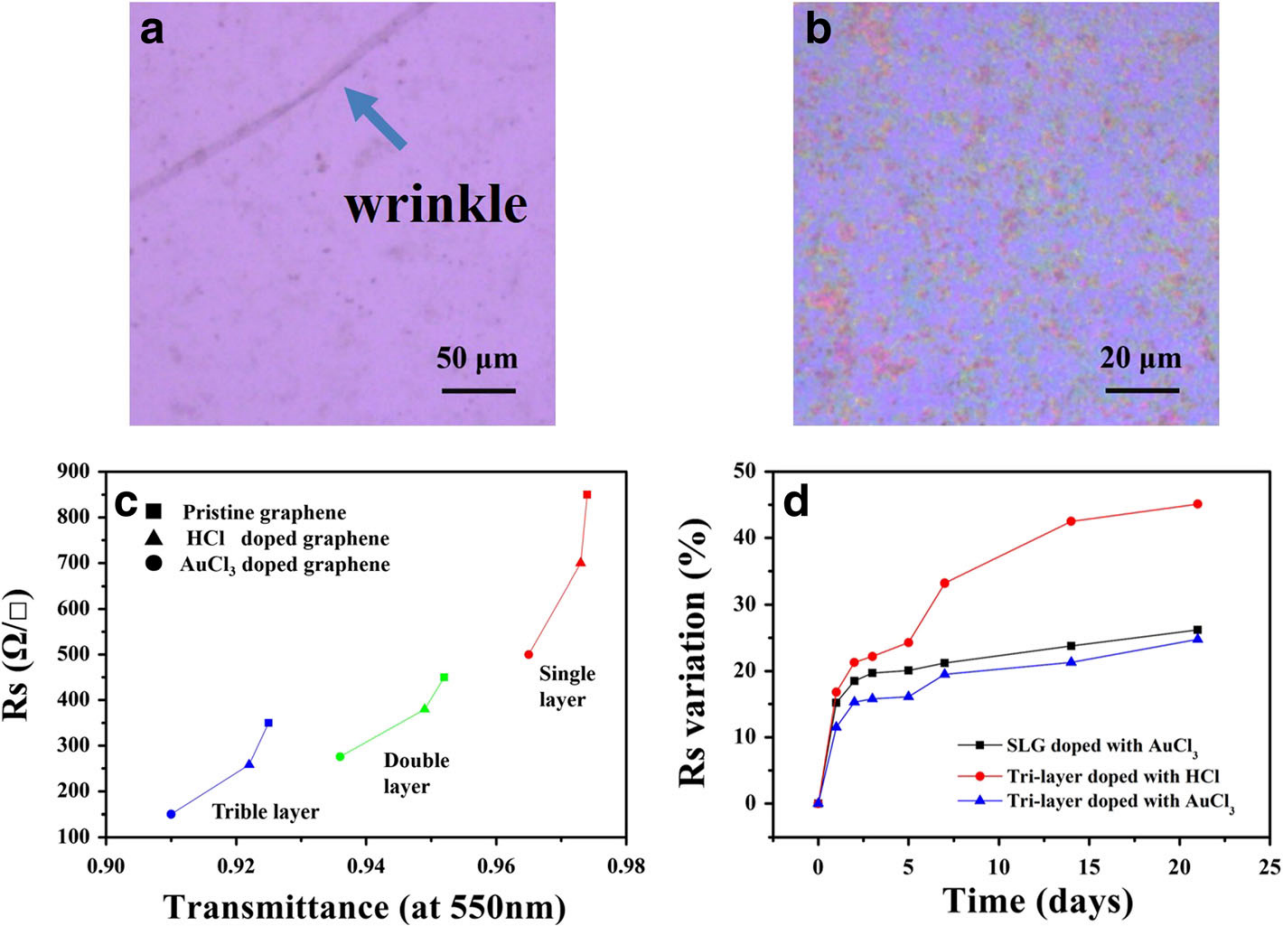
Optical images of **a** the tri-layer graphene film and **b** the tri-layer graphene film doped with AuCl_3_. **c** Sheet resistance and transmittance of the graphene films with different dopants. **d** Variation of the sheet resistance as a function of time for the SLG doped with AuCl_3_, tri-layer graphene doped with HCl, and tri-layer graphene doped with AuCl_3_

Transmittance and sheet resistance are two important indicators for evaluating the transparent conductive electrode. Table 1 and Fig. 3c show the transmittance and sheet resistance of the pristine graphene film and graphene film doped by HCl and AuCl_3_, respectively. Through the lamination and chemical modification, we can see that the sheet resistance of the pristine graphene and the tri-layer graphene modified with HCl and AuCl_3_ are 350, 258, and 150 Ω/□, respectively. The stacking of the graphene layers cannot only reduce the sheet resistance but also lower the transmittance of the films. To maintain the transmittance above 90%, the sheet resistance of the tri-layer graphene is still too high for application. Thus, the chemical modification is necessary to further reduce the sheet resistance. The transmittance of tri-layer graphene is 92.5%, and it dropped to 92.2 and 91% when the tri-layer graphene was doped with HCl and AuCl_3_. The conductivity stability is also an important index to evaluate the graphene film. The significant advantage of AuCl_3_ doping can be demonstrated in Fig. 3d. The tri-layer graphene films doped with AuCl_3_ showed more stability than those doped with HCl due to the volatility of HCl. HCl doping can provide extra holes for the graphene films, which increase the carrier concentration and improve the conductivity [23]. Therefore, the graphene films doped with HCl have lower sheet resistance. We found that the electrical properties of the HCl-doped graphene and pristine graphene tend to be the same after a long time through our research. The decaying effect of HCl doping with time restricts its application in the OLED devices based on HCl-doped graphene films. Fethullah et al. [24] reported that they used spin-coating to achieve layer-by-layer doping, and it showed superior characteristics than topmost layer doping. Compared with their results, our method is more convenient and dispensed in the spin-coating steps. Figure 3a, b depicts the surface profile of the pristine tri-layer graphene film and tri-layer graphene film doped with AuCl_3_.

**Table 1:** Sheet resistance and transmittance at 550 nm of the pristine and doped graphene films, as shown in Fig. 3c

	Pristine graphene		Graphene doping			
			HCl		AuCl_3_	
No. of layers	*T*%	Rs (Ω/□)	*T*%	Rs (Ω/□)	*T*%	Rs (Ω/□)
1	97.4	850	97.3	700	96.5	500
2	95.2	450	94.9	380	93.6	276
3	92.5	350	92.2	258	91.0	150

**Fig. 4:**
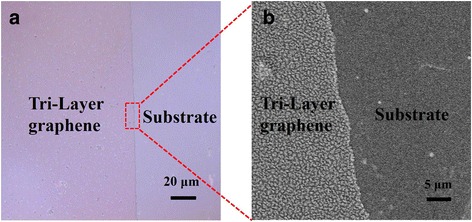
Optical microscope image of **a** the plasma-patterned tri-layer graphene film and SEM image of **b** the edge-enlarged-patterned tri-layer graphene film

**Fig. 5:**
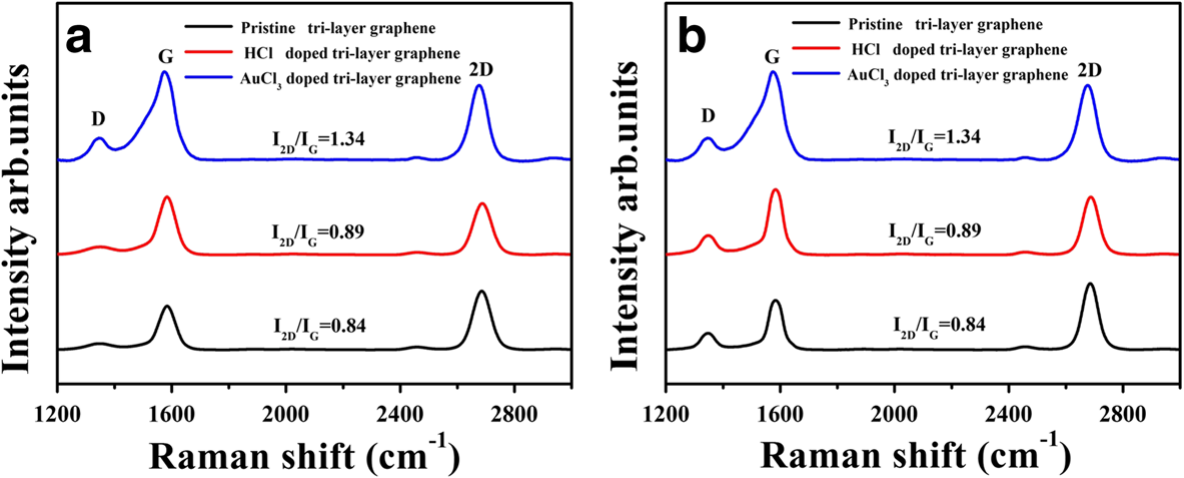
**a** Raman spectrum of the tri-layer graphene with different dopants. **b** Raman spectrum of the tri-layer graphene with different dopants after patterning

The pattern process of the graphene film is critical for the preparation of the OLEDs. We used lithography combined with plasma etching to achieve the pattern of the tri-layer graphene film. Figure 4 shows the boundary which is clear and without burrs of the patterned graphene film. It proves that our graphical method is feasible. Figure 5a shows the Raman spectrum of the tri-layer graphene films with different dopants. We can see that the intensity of D peak of the graphene doped with HCl is lower than that of the graphene doped with AuCl_3_. The reason for this is that the formation of the gold particles between the graphene layer and layer can cause some damage to the structure of graphene film while improving the conductivity of the films at the same time. Moon et al. [22] reported that using laser for patterning graphene caused boundary curl in the graphene films. Our approach avoided this problem. In order to verify the influence of the pattern process on the graphene films, we also did the characterization of the graphene films after patterning. As we can see in Fig. 5b, the intensity of D peak of the tri-layer graphene films doped with different dopants have increased in different degrees. Therefore, the plasma etching process affected the surface roughness of the graphene films, which would cause negative effects on the performance of the OLED devices.

**Fig. 6:**
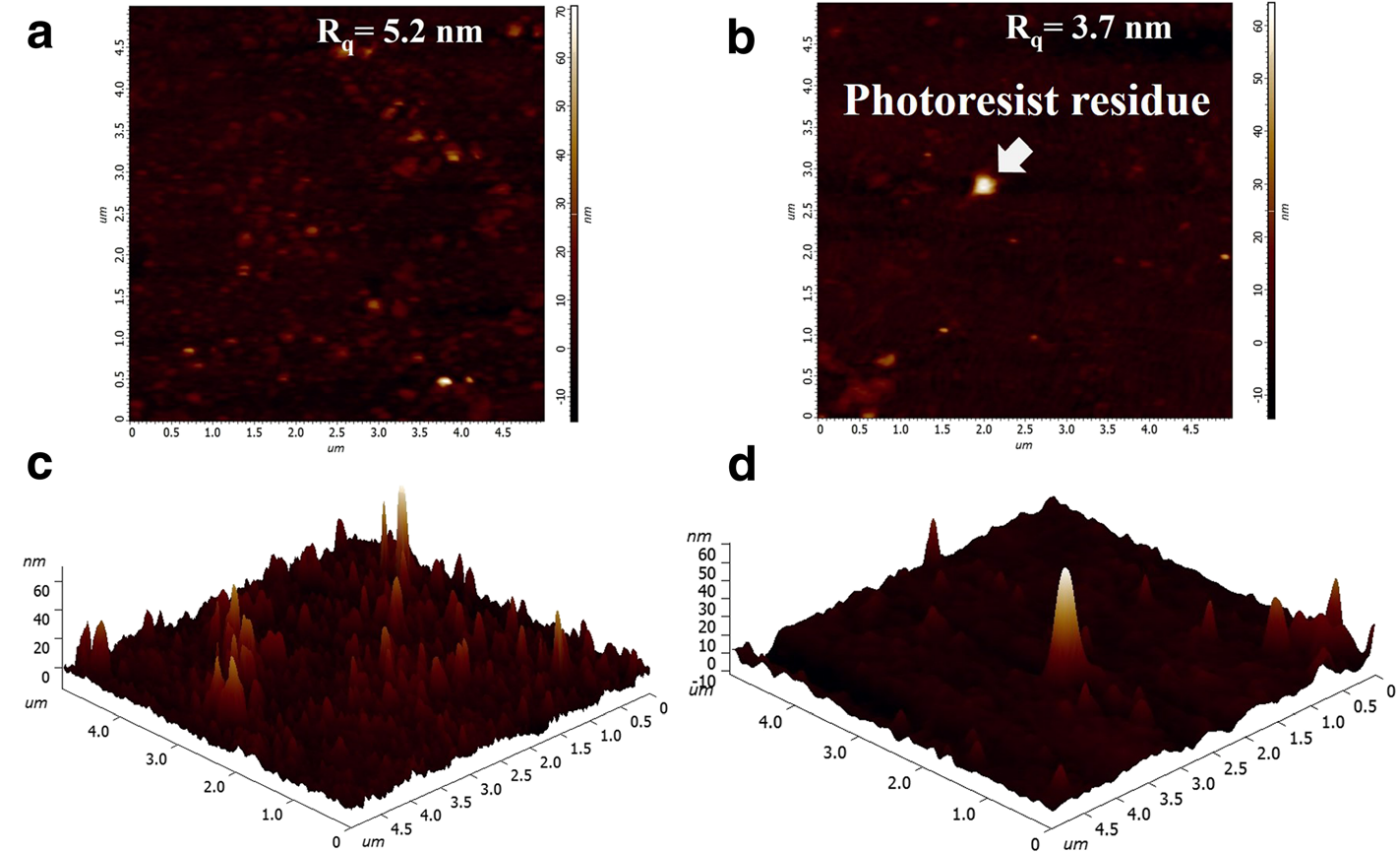
AFM images of the **a** tri-layer graphene film doped with AuCl_3_ and the **b** pristine tri-layer graphene after patterning. **c** and **d** are the 3D displays of **a** and **b**, respectively

The roughness of the film surface is one of the important criteria for judging the OLED anode. Figure 6a, c shows the AFM image and 3D display of the tri-layer graphene doped with AuCl_3_. Fethullah et al. [24] reported that doping AuCl_3_ can form Au clusters to affect the RMS (root mean square) of the graphene films, which is similar with our results. The RMS of the tri-layer graphene doped with AuCl_3_ is 5.2 nm, which is higher than the pristine tri-layer graphene after patterning. From Fig. 6b, d, we also can see that the lithography process has left the photoresist residue on the surface of the graphene film. In the process of etching graphene films, photoresist was used as the protective mask. The process of plasma etching will cause changes in the photoresist at the molecular level, which makes it difficult to remove the photoresist entirely by acetone. Therefore, photoresists tend to leave a thin residue on the graphene surface and affect the roughness of the graphene film. Meanwhile, the photolithography process can also lead to mechanical damages to the graphene by the stress when the resist was stripped. It is necessary to further study on the photolithography process of graphene films and its influence on the performance of the OLEDs based on graphene anodes.

### Characterization of the OLEDs

**Fig. 7:**
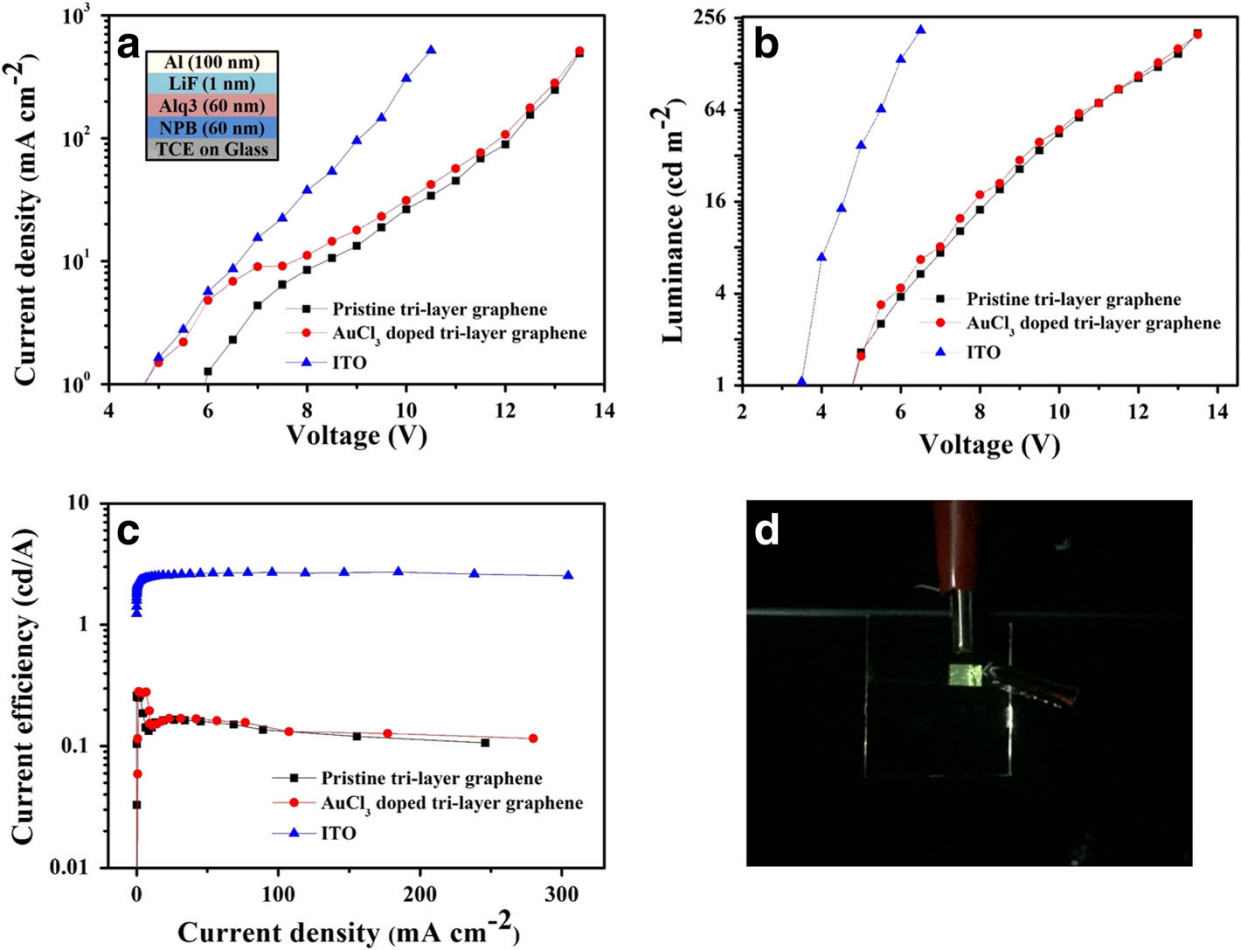
Structure of OLED (*inset*) and **a** current density–voltage, **b** luminance–voltage, and **c** current efficiency–current density characteristics of the ITO, tri-layer graphene, and the tri-layer doped graphene-based OLEDs. **d** The photograph of an emitting device based on the tri-layer graphene doped with AuCl_3_ anode

The OLEDs with the anode of the tri-layer graphene and tri-layer doped graphene have the structure of anode/NPB (60 nm)/Alq3 (60 nm)/LiF (1 nm)/Al (80 nm). The OLEDs based on the ITO anodes were also fabricated for comparison. Due to the electrical performance of tri-layer graphene doped with HCl and pristine tri-layer graphene tending to be consistent after a long time from Fig. 3d, we omitted the fabrication of OLEDs based on the tri-layer graphene doped with HCl anodes. Figure 7 shows the current density–voltage characteristics, luminance–voltage, and the current efficiency–current density characteristics of the devices, respectively. We can see that the current density and luminance at each specific drive voltage of the devices based on the tri-layer graphene and doped graphene exhibit lower values than those based on ITO. Meanwhile, the devices based on the tri-layer graphene and tri-layer-doped graphene show a higher turn-on voltage of 4.8 V than those based on ITO (3.5 V). It is due to the higher sheet resistance of the tri-layer graphene (~300 Ω/□) and the tri-layer graphene doped with AuCl_3_(~150 Ω/□) composite films than that of ITO (~20 Ω/□). The current efficiency of the two devices are lower than those of the ITO-based device. The best device in our experiment based on the tri-layer graphene doped with AuCl_3_ anode exhibits a current efficiency of 0.263 cd/A. There are four main reasons for the poor performance of the devices: (1) the most important factor is that the work function of graphene (about 4.4 eV) is lower than that of the ITO (about 4.8–5.0 eV) so that the hole injection barrier of tri-layer graphene is larger than that of the ITO and finally affected the carrier injection; (2) the conductivity of the graphene films is very poor, which affects the injection of carriers; (3) the process of the patterning compromised the quality of the graphene films; and (4) the influence of surface roughness on the graphene anodes. The simple device structure is also a possible reason of the poor device performance. Figure 7d shows the photograph of the OLED device based on the tri-layer graphene doped with AuCl_3_ anode at 150 cd/m^2^. The hole injection layer (HIL) can enhance the current of holes and hole injection efficiency toward the emission zone [25–27]. In order to improve the performance of the OLEDs based on graphene film anode, a HIL such as PEDOT:PSS can be inserted between the anode and NPB.

## Conclusions

In conclusion, we developed a new method to prepare tri-layer graphene films with chemical modification and explored the influence of doping and patterning process on the performance for the graphene films as transparent electrodes. We have demonstrated that the tri-layer graphene films can be used as transparent and conductive anodes in the OLEDs. The performance of the devices based on the tri-layer graphene films doped with AuCl_3_ is higher than that of the devices based on the tri-layer pristine graphene film due to the contribution of high conductivity. These results suggest that the tri-layer-doped graphene films are a promising candidate as the transparent conductive electrode in optoelectronic devices.
